# Accelerating the Morphogenetic Cycle of the Viral Vector *Aedes aegypti* Larvae for Faster Larvicidal Bioassays

**DOI:** 10.1155/2020/7405421

**Published:** 2020-08-29

**Authors:** José Domingos Fontana, Rafael Lopes Ferreira, Tatiana Zuccolotto, Cibelle de Borba Dallagassa, Leonardo Pellizzari Wielewski, Barbara Maria Santano Chalcoski, Mario Antonio Navarro da Silva, Vinicius Sobrinho Richardi, Jonas Golart, Cynara de Melo Rodovalho

**Affiliations:** ^1^Master Program on Urban and Industrial Environments, Federal University of Paraná (MAUI-UFPR), Curitiba, PR, Brazil; ^2^Graduate Program in Environmental Science and Technology, Academic Department of Chemistry and Biology, Federal Technological University of Paraná (UTFPR), Curitiba, PR, Brazil; ^3^Department of Pharmacy, Federal University of Paraná (UFPR), Curitiba, PR, Brazil; ^4^Culicidae and Chironomidae Morphology and Physiology Laboratory, Department of Zoology, Federal University of Paraná (UFPR), Curitiba, PR, Brazil; ^5^Arthropod Vectors Control and Physiology Laboratory, Oswaldo Cruz Institute (FIOCRUZ), Manguinhos, Rio de Janeiro, RJ, Brazil

## Abstract

Any bioassay to test new chemically synthesized larvicides or phytolarvicides against *Culicidae* and more harmful mosquito species, such as *Aede*s *aegypti* and *Aedes albopictus*, which specifically transmit dengue, yellow fever, chikungunya viral fevers as well as Zika virus, or *Anopheles gambiae*, a vector for malaria and philariasis, requires thousands of well-developed larvae, preferably at the fourth instar stage. The natural morphogenetic cycle of *Aedes* spp., in the field or in the laboratory, may extend to 19 days at room temperature (e.g., 25°C) from the first permanent contact between viable eggs and water and the last stage of larval growth or metamorphosis into flying adults. Thus, accelerated sequential molting is desirable for swifter bioassays of larvicides. We achieved this goal in *Aedes aegypti* with very limited strategic and low-cost additions to food, such as coconut water, milk or its casein, yeast extract, and to a lesser extent, glycerol. The naturally rich coconut water was excellent for quickly attaining the population of instar IV larvae, the most advanced one before pupation, saving about a week, for subsequent larvicidal bioassays. Diluted milk, as another food source, allowed an even faster final ecdysis and adults are useful for mosquito taxonomical purpose.

## 1. Introduction

Outbreaks of viral fevers such as dengue, chikungunya, yellow fever, and particularly Zika threaten public health in many tropical and subtropical countries. *Aedes aegypti* and *Ae. albopictus* are most frequently implicated as the virus vectors, followed by *Anopheles* spp., protozoan and nematoid transmitters. The real risk that pregnant women might be infected with Zika and give birth to microcephalic infants has generated panic in the population, especially in northeastern Brazil. In addition, a recent report found that the most common domestic mosquito *Culex quinquefasciatus* may also transmit the Zika virus [1], although another research disagrees with this assertion [2].

The reports and warnings of health authorities are frightening. The distribution and evils of dengue and related fevers as evaluated some years ago have become even more severe. Upon a modeling with a 95% credible interval, 390 million dengue virus infections are estimated worldwide by the World Health Organization (WHO) [3]. In Brazil, according to the official reports from the National Health Ministry, the cases of *Aedes* spp.-transmitted arbovirus was 265,900 to 2,527,000 cases for dengue fever with 754 deaths; 87,700 to 130,800 cases for chikungunya; and 8,700 to 10,700 cases of Zika virus, between 2018 and 2019 [4].

In some Brazilian states, monkeys are still being infected and dying from yellow fever as reported by Andre Duchiade in the magazine Scientific American from July 5, 2018. Health authorities have accepted that mosquito control must utilize integrated pest management (IMP). When there is a clear risk that an outbreak will evolve from an endemic to a pandemic, drastic actions are taken to control mosquitoes with powerful synthetic insecticides, despite progressive environmental accumulation and negative alterations in the nervous system of humans and animals. These chemicals include the synthetic pyrethroids and temephos (Abate, BASF), an organophosphate larvicide. Mosquito repellant is another strategy, along with media alerts and distributing pamphlets to the population [5].

Both male and female adult mosquitoes display a preference for flower nectar because it provides a quick source of energy through simple sugars, as well as protein. However, a newly emerged female will mate and desperately seek human or animal blood, since hemoglobin is hydrolyzed in the gut of the female mosquito and provides free amino acids and strategic iron for the new cycle of egg laying and eclosion [6].

The larval cycle is an ideal time to combat mosquitoes, developing inside or outside homes in saucers underneath plants, abandoned tires, or uncovered water storage tanks, which can be treated with chemically synthesized larvicides or, preferably, phytolarvicides.

To avoid accumulation and damage from synthetic insecticides, researchers around the world are attempting to develop new and more innocuous phytolarvicides. A recent review [7] lists 30 different plant extracts that are considered as lethal to *Aedes* spp. and *Anopheles* spp., thus encouraging the utilization of these safer tools to replace biocides from chemical synthesis and their harmful effects on soil, water, and vegetable foods.

Public health laboratories have a permanent need for thousands of mature larvae for each larvicidal bioassay either utilizing novel or known synthetic insecticides or safer natural products.

The WHO guidelines for larvicidal bioassay screening and efficacy require at least 4 × 20 larvae for each assayed dose of a new substance and between 6 and 10 different and progressive doses to estimate the usual LC_50_ value (lethal concentration to kill half the larval load). Ideally, these bioassays should be conducted on instar IV larvae and require 480–800 larvae to complete each assay for each natural or synthetic insecticide. The time needed to reach the appropriate stage of larval development presents a challenge. Even in optimized laboratory conditions, an average of 8–18 days is required, but this period depends on food supply and temperature (ranging from 10.4°C to 29.7°C). One study on *Ae. albopictus* in the Indian Ocean region used a progressive daily feeding platform based on yeast extract and found the following average periods for each ecdysis stage: 5.6 days for egg hatching, 1.8 days for instar I to II, 1.3 days for instar II to III, 1.9 days for instar III to IV, 3.2 days for instar IV to pupa, and 2.3 days for pupa hatching period [8, 9]. Other reports mention longer time courses [28, 30].

Comparing instar IV larvae with any previous morphogenetic stage from egg to instar III larvae, the instar IV clearly has an advantage over early instars, under a magnifying glass, to observe and discriminate anatomic and morphological features for trustable mosquito identification. As an example, the thorax segment VII scale combs are a clearly distinguishable feature between *Ae. aegypti* and *Ae. Albopictus*, since in the former these pair of accessories (one in each side of the thorax) have large medial spines rounded by stout subapical spines, which are absent from the latter [10].

The design of the scutum/scutellum, which is a white lyre-shaped marking with 4 lines on *Ae. aegypti* but is a single median longitudinal white line on *Ae. Albopictus*, is more clearly visible on the dorsal surface of adult mosquitoes (e.g., those evolving quicker from milk-fed larvae) [11].

Even the natatory behavior of two somewhat unrelated mosquitoes, *Aedes* (virus vector) and *Anopheles* (protozoan transmitter), more easily observed by naked eyes in the larger instar IV larvae, shows a marked differential feature at each resting time interval: *Aedes* holds the body vertically to the water surface while *Anopheles* aligns horizontally.

There are many other anatomic and morphological aspects between *Aedes* species (including those reported to be of no medical interest), including some specific for female larva and adults, that can be distinguished through a laboratory assignment, which is, again, facilitated with instar IV larvae or adults quickly obtained according to the food intakes herein described [12, 13].

Some researchers prefer not to change the water during the mosquito growth cycle, because growing larvae are able to reprocess their excreta, supplying their minimal needs for carbon and nitrogen, while convenient microflora such as mutualist bacteria can continue their parallel growth. This latter situation may be common since (a) fresh eggs may be collected that have microorganisms in their outer shell and (b) boiled and reaerated tap water for complete dechlorination can come into contact with nonfiltered air (e.g., open trays for egg hatching; permanent air flow over the bioassay cups). It is our experience that tap water dechlorinated by boiling and aeration with an aquarium pump provides approximately 19.3 mg of salts per liter gravimetrically measured through lyophilization or drying in a ventilated oven at 110°C for 20 min. Thus, some salted food supply must be considered even in the bioassay controls.

This study investigated the use of low-cost food sources to hasten development of newly hatched larvae of *Ae. aegypti* (first instar) to the fourth instar stage to produce larvae more rapidly for testing larvicides. These sources included fresh coconut water, several types of cow's milk and its casein-derived fraction, and yeast extract, and, less often, the very simple polyol, glycerol. Obtaining significant results can save time for subsequent natural or synthetic larvicide bioassays as well as mosquitoes with trustable taxonomy (although not included in this study), with faster production of adults.

## 2. Materials and Methods

### 2.1. Source of *Ae. aegypti* Eggs and Larvae

Eggs from the noninfected Rockefeller strain of *Ae. aegypti* were obtained from the Vector Arthropod Physiology and Control Laboratory at the Fundação Oswaldo Cruz (FIOCRUZ; Rio de Janeiro, Brazil). Eggs in amounts varying from 1,500 to 3,000 were transferred from the dry filter paper strips to dechlorinated tap water (previously boiled and aerated) in an open 2 L container and kept in a BOD incubator at 25°C ± 0.5°C in an alternating 12 h dark/light cycle until hatching occurred. The obtained instar I larvae were then utilized in the quadruplicated experiments with various nutritional sources maintaining treated tap water as controls.

### 2.2. Nutritional Sources

The nonpowdered cow's milk was purchased from Nestlé Brazil, and soy milk was purchased from Alpro, Belgium. They were diluted 1 : 10 to provide the desired final concentration. Normalized addition of each milk was calculated from the total solid weight obtained from lyophilization of the parent liquid. The three types of cow's milk were fractionated by isoelectric precipitation of casein through careful addition of diluted hydrochloric acid until pH 4.7 was reached, followed by centrifugation and casein precipitate recovery, and then reconstituted to the parent volume and pH.

Coconut water was aseptically extracted from fresh green and mature coconuts (*Cocos nucifera*), purchased at a local market, and maintained frozen until use as 1 : 10 dilutions. Glycerol (85%) was obtained from Reagen Co. and dry yeast extract from Sigma-Aldrich, and both were prepared, respectively, as concentrated mother solutions (1 g%), sterilized by three cycles of incipient boiling in a microwave oven, and then kept frozen until use.

As parallel controls for the carbohydrate and/or energy supply contained in glycerol, coconut water, and whole milk, sterilized solutions of 2 mg/mL glucosamine, N-acetylglucosamine, chitosan, and chitin were also prepared for the prospection of their positive effect in the *de novo* biosynthesis of chitin.

### 2.3. Bioassay of Nutrients

For all nutrient effects on *Ae. aegypti* morphogenetic cycle progress, we followed our previously published methodology [14], including statistical details. Briefly, 20 first instar larvae as quadruples were placed into each 70 mL PET transparent plastic container with the respective liquid (control or experimental treatments as 35 mL final volume). The lid of each container was pierced 4 times for sufficient gas interchange, but not large enough for an adult mosquito to escape. The control groups were maintained in the same water (W) that they hatched from. Feeding regime is described in Table 1, and *Ae. aegypti* growth was followed until the seventh day at 25°C. Development through the four instars, pupal stage, and adult emergence was monitored and recorded daily. Once the bioassays were finished, all larval forms, pupae, and adults were collected in a stainless 200 mesh screen and crushed with the help of a rigid silicon spatula. In the case of flying adults and to avoid the risk of air dissemination, their containers were previously saturated with ethyl acetate to kill them. The media pH on the seventh day was measured with a mini-pH meter from Akso (Simpla PH140).

### 2.4. Monitoring of Mutualist Bacteria

The presence and contribution of bacterial microflora, which developed alongside the eggs or hatched larvae, in the two milk sources as food supply, were assessed in 1.5 mL samples, taken from the whole milk (WM) and reduced-fat milk (LM) larvae-free bioassays on the seventh day, which were centrifuged in a spin centrifuge at 10,000 × *g*. Bacterial pellets were spread in Muller-Hinton's agar dishes incubated at 30°C for 48 h and then subjected to Gram staining.

### 2.5. Phytolarvicide Bioassays

To validate the normal physiology of instar IV larvae following the acceleration of the morphogenetic cycle, they were challenged and killed with piperine/isobutylamides from the whole ethanolic extract of *Piper nigrum* fruits (black pepper) to confirm our previously found LC_50_ [14]. Briefly, whole, mature, and dried fruits of black pepper were triturated in a blender following addition of 10 vol. of absolute ethanol and heating to incipient boiling. The filtered extract was concentrated under vacuum to a deep green paste, and a stock solution was prepared from this to ensure, after dilutions, a range from 0.5 to 25 ppm for *Ae. aegypti* (instar IV larvae) phytolarvicidal bioassays.

### 2.6. Statistical Analyses

Experimental data concerning mortality percentages by black pepper phytolarvicides were calculated by the PROBIT method [15] and means, standard errors, statistical significance, and histogram drawings for all *Ae. aegypti* body forms resulting from feeding on different nutrient sources were processed by the two-way ANOVA followed by the Tukey post hoc using the Prism 6 software from GraphPad Prism Software Inc. Other details on this subject may be found in our previous report [14].

## 3. Results

**Figure 1:**
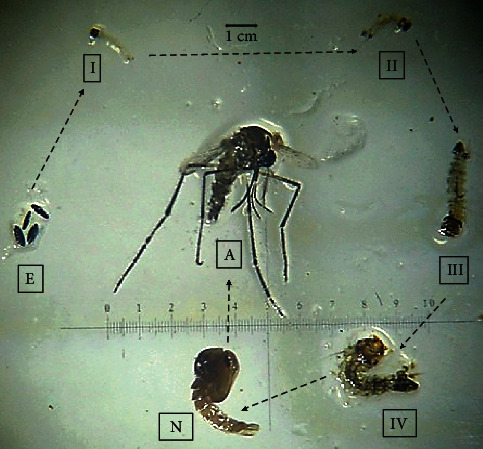
The complete morphogenetic cycle of *Aedes aegypti* grown in fortified milk from daily sampling from zero to 240 h at 25°C and light-dark cycles of 12 h.

For a better understanding of the anatomy and morphology of the different and progressive forms of *Ae. aegypti* in its molting cycle, Figure 1 depicts these anatomic and morphological details.

Taking into account that chitin is the major macromolecule in all stages of *Aedes* development, our first effort was the isolated addition of glucosamine, N-acetyl-glucosamine, chitin, or chitosan (all normalized to 2 mg/mL of tap water) in order to tentatively induce or enhance chitin biosynthesis Surprisingly, no net benefit was obtained to shorten the mosquitoes' growth time. Additionally, the exuviae or the sequentially discarded remains of the chitinous skeleton, as microscopically observed, remained intact. No chitinolytic activity was detected in a mix homogenate of instars. Hence, these were clues that the permanent biosynthesis of chitin was utilizing other sources for the glucosamine building block. Any contribution of chitinase arising from bacterial contamination/symbiosis could not be ruled out, especially in bioassays supplied with milk. In fact, larvae-free culture media, inoculated in the nutritional Muller-Hinton media, led to marked bacterial growth, for instance of Gram+ cocci.

### 3.1. Effect of pH on Ecdysis

Some gains in the ecdysis rate may derive from the hydrogenionic potential of the growth media. Some significant variations in pH were observed when the third instar larvae transitioned to the fourth instar, dropping from pH 8.2 to 7.9 in the control (tap water) and even to 4.0 in the milk samples, probably due to mutualist bacteria in the liquid medium and then fermenting free sugars. Cultures in milk, although diluted, emitted an intense lactic acid odor.

### 3.2. Effect of Nutrient Sources in Accelerating the Morphogenetic Cycle

**Figure 2:**
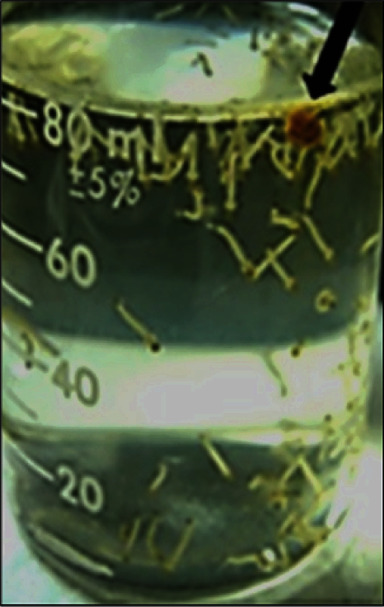
Solid yeast extract as an attractant for instar IV larvae of *Aedes aegypti.*

Third and fourth instar larvae of *Ae. aegypti* agglomerated in dense clusters when the container was illuminated from the side, which facilitated capture for bioassays. Since yeast extract proved to be the source with the more pronounced smell and taste, a grain sample was placed in an instar IV larvae population fasted for 24 h. As illustrated in Figure 2, they quickly swam around and took the offered food thus confirming the refined sensatory apparatus of mosquitoes. Probably, the peculiar smell/flavor of this extract explains this behavior of the larvae. *Aedes* can assimilate a strategic food supply even from intact yeast cells as the sole nutritional source thanks to the presence of *β*-glucanase in the mosquito head, gut, and the entire body [16].

Milk can support mammals in their first year as the sole source of food, due to the rich nutritional composition encompassing casein, lactoglobulins, lactose, and salts. As a phosphoprotein, casein also provides valuable input of phosphorus and calcium. Open or lid-pierced plastic vessels were used for the bioassays. Therefore, the observed lactic smell of fermented milk may be either from airborne lactic acid bacteria or mosquito gut microflora fermenting the milk substrate. The alternative nutrition, regulatory, or energy sources were, respectively, coconut water, yeast extract, and glycerol. A single combination of the second and third sources was also bioassayed for synergism, with no encouraging results found.

**Figure 3:**
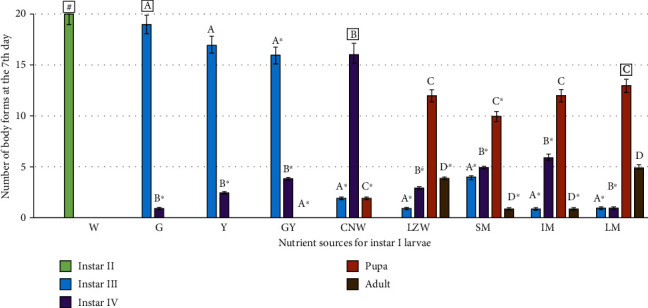
Quantitation of all mosquito forms at the seventh day of the *Ae. aegypti* morphogenetic cycle as a function of the nutrient sources (# = instar II; a = instar III; b = instar IV; c = pupa; d = adult; W = water; G = glycerol; Y = yeast extract; GY = glycerol + yeast extract; CNW = coconut water; LZM = lactose-free milk; SM = soy milk; WM = whole milk; LM = reduced-fat milk).

Figure 3 summarizes the main results for the acceleration of the *Ae. aegypti* morphogenetic cycle or its successive ecdysis as a function of nutrient inputs after 7 days of incubation at 25°C.

The framed letters in Figure 3 designate the dominant body form amongst all and resulting from any particular nutrient source. Any repeated letter indicates no statistical difference from the reference one indicated by the frame. Any repeated letters with an asterisk indicate statistical difference from the dominant body form (Tukey's test; *p* < 0.005).

Control or incubation in water (Figure 3; “W” and “#”) allowed a uniform and single advancement of instar I larvae just to the next step or instar II. Glycerol (Figure 3; “G”) and yeast extract (Figure 3; “Y”) allowed a second marked larvae advancement to instar III (Figure 3; “a” or “a∗”) and their combination (Figure 3; “GY”) did not lead to synergism suggesting that the best benefit originated from the second. Coconut water (Figure 3; “CNW”) allowed the main goal to be attained, since 80% of the larvae population (Figure 3; “[b]”) reached the desired instar IV as compared with just 25% or 30% (Figure 3; “b∗”) in the SM and LM milks, respectively. Further evolution of instar IV larvae into pupae was marked in any of the four milks offered as food (Figure 3; “c”, “c∗,” and “[c]”) as this form always exceeded 50% of each whole population. Furthermore, LZM and LM milks also led to the generation of the most advanced mosquito morphological and functional form, the flying adults as 20 and 25% of all forms, respectively (Figure 3; “d” and “d∗”). Among the 6 food categories for feeding *Aedes aegypti* larvae, skim milk contains the most animal proteins along with many other nutrients. However, no further details were provided by that author whose sole interest was the negative effect of scum on breeding mosquitoes [17].

The average total dry solids of these milk contents varied from 14 to 22 mg/mL, which is higher than the literature reports [18, 19] with average values of 12.5–13.7 g% total solids and 3.8–4.6 g% total fat, 3.1–5.3 g% protein, 4.0–4.6 g% lactose, and 0.4–0.8 g% of salts (as ashes), depending on animal breeds and feeding regime. Their use was normalized, after dilution, to 1.4 mg/mL.

As compared to glycerol, a simple hydroxylated C skeleton, yeast extract is, by far, a richer mixture of nutrients including amino acids, peptides, sugars, nucleotides, vitamins, and salts. Hence, it also sustains, through other biochemical pathways, the molting steps of *Aedes*. A similarly complex extract is usually prepared from yeasts following autolysis and/or enzymatic treatment of whole cells. The average composition of the main components of yeast extract is 52% for proteins, 35% for soluble carbohydrates, 7% for salts, 5.5% for lipids, and a minor percentage of nucleotides and vitamins [20, 21].

Coconut water, on average containing around 6 g% of total solids including [22, 23] 3 g% sucrose, 1.2 g% glucose, 0.8 g% fructose, 0.51 g% fats, free amino acids, organic acid, vitamins, and 0.43 g% of salts (as ashes), has a more complex energetic and nutritional composition than any of the other feeding sources herein employed. Hence, CNW efficiently supported and anticipated development of mosquito larvae to the desirable instar IV stage at a 1 : 10 dilution. Coconut water saved around one week to attain instar IV larvae from freshly hatched (instar I) *Ae. aegypti larvae* compared to other literature data from *Aedes albopictus*, a closely related species to *Ae. aegypti* [24].

All four milk samples diluted 10 times led to dominant 50 to 68% pupation of the larvae (Figure 3; “c,” “c∗,” and “[c]”), a result not directly pursued in this report. A parallel inconvenience was the evolution of part of the pupae into flying adults, which required more stringent laboratory care like pierced plastic lids for the bioassay cups.

For the specific purpose of this study, namely, a rich supply of instar IV larvae, results obtained with coconut water and, secondarily, with isoelectrically precipitated milk casein proved relevant; in the case of the former, at 5 *μ*g/mL, 4/5 of all forms were larvae at stage IV as shown in Figure 3 (“CNW”) in its central column (“[b]”). The equivalent result for purified casein was in the 2/3 to 3/4% range, depending on the milk source.

In the controls—dechlorinated/aerated tap water with or without 1% ethanol—all 20 larvae were stuck in the second stage or instar II on the seventh day, a foreshadowing that their complete molting towards flying adults would take longer than two weeks.

Taking into account the more positive effect of coconut water, a further exploration of laboratory-available nutritional sources was also carried out focusing on any additional concentration-derived benefit. Yeast extract (although the most expensive nutrition source), glycerol, and peptone were again bioassayed at a higher concentration (0.25%) as well as a larger incubation (10 days). Most of the larvae attained instar III and IV in the case of the latter two sources, but the best benefit arose from the first source since the attained ratios for instar IV pupae in the quadruplicates were 14/16/14/15 : 6/4/6/5 (SD ± 0.95743). The latter bioassays and the narrow data variations as equally observed in data from Figure 3 provided satisfactory support for the whole results from a statistical view point.

**Figure 4:**
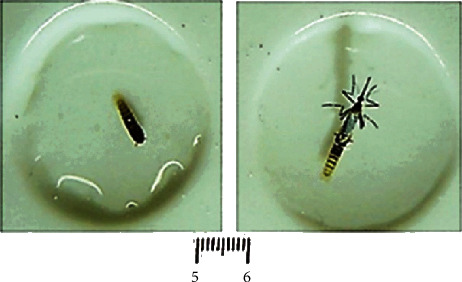
An adult *Aedes aegypti* on delivery from its mature pupa completing the mosquito morphological and physiological cycle.

Mature pupae swim in ellipsoid movements and are faster than the larvae in any instar, alternating with still moments. Just before an adult emerges, the pupa remains immobile. Complete adult emergence from the mature pupa is rapid, lasting just a few seconds.  Figure 4 shows these two forms, which complete the morphogenetic cycle of *Ae. aegypti*.

### 3.3. Phytolarvicidal Bioassay against Precociously Produced Instar IV Larvae of *Ae. aegypti*

The normal physiology was confirmed for the early larvae obtained with the supply of strategic food sources like coconut water. An initial approach was to monitor the transition of pupae (reared in coconut water) to flying adults as shown in Figure 4.

**Figure 5:**
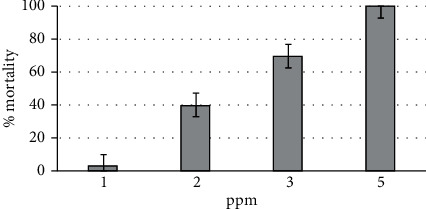
Mortality percentages ± SE of *Aedes aegypti* instar IV larvae reared in coconut water killed by crude ethanolic extract of *Piper nigrum* fruit.

Beyond this, we demonstrated that the acceleration of the morphogenetic cycle of *Ae. aegypti* with the tested diets did not alter the susceptibility of the resulting larvae when challenged with phytolarvicides. The WHO protocol, which we have previously used [14], was repeated to test three different batches of raw ethanol extracts of air-dried *Piper nigrum* fruits, which we already reported as being one of the most potent mix of phytolarvicides against *Aedes* spp. This ethanol extract was used against instar IV larvae, which had been reared on coconut water. As indicated in Figure 5, the resulting LC_50_ for the black pepper extract, just above 2.0 ppm, did not significantly differ from our previous data (LC_50_ = 1.84 ppm) found in a study investigating this extract with regard to phytolarvicidal synergism between black pepper piperine/isobutylamides and soursoap acetogenins when ethanol extracts from *P. nigrum* fruits and *Annona muricata* seeds were blended [14].

## 4. Discussion

Concerning our choice of advanced instar for larval collection, among the progressive morphogenetic cycle, instar IV larvae are naturally the more advanced in their growth and physiology of the 4 ecdysis steps preceding pupation: (a) Both of the above parameters directly depend on the ability of food digestion, and only instar IV has both forms of the strongest proteolytic enzymes (trypsins) [25]. (b) Instar IV larvae generally spend more time actively foraging than previous instars and hence have improved uptake of food offered in the nutrient medium [26]. *Ae. aegypti* eggs are 1 mm or less and barely visible to the naked eye in clean water. However, reared larvae even in the first stage or instar I are easy to see as they swim vigorously. After several days, the largest fourth instar is achieved, reaching 0.5 cm and continuing to swim until the pupal stage begins. *Aedes* larvae, irrespective of the instar, almost continuously swim except for intervals when they align their siphons in the water-air interface to renew their oxygen supply [27]. Thus, thanks to this visibility, the larval cycle, especially the IV instar, is an ideal time to combat mosquitoes before they become flying hard-to-control adults.

With respect to the complete morphogenetic cycle and prospection on *de novo* biosynthesis of chitin during the growth and differentiation of *Ae. aegypti* growth, as shown in Figure 1, it starts from eclosion; encompasses four larval instars, the pupal stage, and flying adult; and involves repeated ecdysis or molting stages to accommodate the growing mosquitoes. As they discard and replace their chitinous exoskeleton, the insects intensively produce chitin, the main macromolecular component of the cuticle, followed by melanin, a polymerized form of oxidized tyrosine. The carbon and nitrogen sources used for this dynamic anabolism are not clearly understood. The excreta of the growing larva are thought to play an important role in supplying these elements, but the larval gut microflora may also be a source. All these aspects were reviewed in depth in an encyclopedic book on *Aedes aegypti* [27].

Mutualist bacteria were present in our culture media, mainly in those where milk was utilized as a food source probably due to the large open tray for hatching eggs kept inside an incubator chamber with permanent circulation of nonsterilized air.

For the purpose of this study, the results obtained just with coconut water or isoelectrically precipitated casein instead of whole milk are relevant given that the yield attained for instar IV larvae is desirable for further bioassays of the larvicidal effect of synthetic insecticides and, more preferably, for environmentally safer phytolarvicides. Feeding of whole milk to instar I larvae also proved of interest for a parallel purpose, namely, the secure identification of *Aedes* species from flying adults.

The literature contains limited information about intentional manipulation of *Ae. aegypti* development through specific foods or nutrients. Pupation time of *Ae. aegypti*, at 26°C, with a progressive diet on fish meal, sucrose, and finally blood, was between 15.5 and 21 days in most of the experiments with no significant difference between males and females. The time was shorter, between 10 and 19 days, for *Ae. albopictus* and somewhat slower in males [28]. A report [29] on the molting of *Aedes* (fourth instar to pupa) found a drop in juvenile hormone while ecdysone increased. Food limitation or restriction at this stage impeded pupation, unless a previous condition of marked food/energy reserve was introduced at the larval stage. Levi et al. [29] used Pharmamedia (ADM), a finely ground yellow flour containing the native globular proteins of cotton seed embryos, and obtained the following ecdysis times for each stage: first to second instar: 1.0–1.4 days; second to third instar: 1.1–3.2 days; third to fourth instar: 2.1–4.3 days; fourth instar to pupa: 4.0–4.5 days; and pupa to adult: 5.6–6.0 days. They found that Pharmamedia supply for neonate larvae at 0.4 mg/mL led to the shortest time for larvae to reach instar IV. However, most concentrations of this particular supply also led to reduced larval survival. Conversely, the diets these authors have explored led to no larvae mortality.

In another report, with a 1 g% aqueous diet mixture composed of beef-liver powder, tuna meal, and vitamin mix, the whole larval stage of *Aedes aegypti* lasted 16.57 days at 24°C and 19.19 days from hatching to flying adults under the same conditions [30].

With respect to *Ae. albopictus*, the development up to the fourth instar was strongly influenced by the temperature, for example 14.48 days at 20°C [9, 17].

Our results are even faster; at 7 days, 80% of larvae reached the desired instar IV stage, using simpler and less expensive food sources such as coconut water (or laboratory-prepared milk casein), as shown in Figure 3 (“CNW” and “[b]”). The rate of 80% viable larvae after eggs hatched is acceptable according the Food and Agriculture Organization of the United Nations.

The belief that *Aedes* spp., which harbor and transmit harmful viruses (namely, *Ae. aegypti* and *Ae. albopictus*), preferably or exclusively seek out and deposit their eggs in clean water was investigated [31], and it was found that *Aedes* spp. will lay eggs in plastic/ceramic containers or even in sewers. Although eggs are less frequently deposited in sewers, the resulting pupae present greater biomass and the adults have undoubtedly a double advantage for survival.

Since research has clearly established that both species of *Aedes* (*aegypti* and *albopictus*) are vectors for the four main viral fevers as well as Zika virus, interest in their feeding preferences is timely. An intensive study investigated the growth performance of both *Ae. aegypti* and *Ae. albopictus*, respectively, collected in Tanzania and Japan fed with a mixture of rat food and yeast extract at low and high nutrient input. At 25°C and with a low food input of 50 *μ*g daily, the entire morphogenetic cycle (from hatching till emergence) of *Ae. aegypti* took 14 and 19 days, respectively, for male and female insects [32]. We have been using a far more reduced food supply and have given food just once to the instar I larvae (e.g., 6 *μ*g/mL for coconut water; 16.6 *μ*g/mL for yeast extract or glycerol).

Sucrose (10 g%) produced longevity in *Aedes* females and males at the same or greater rate than citrated hemolyzed beef blood or an alternative synthetic mix of amino acids, inorganic salts, and 10% sucrose [33]. Sucrose, obviously, is an interesting energy source for newborn females when visiting flowers for nectar.

Our findings led us to investigate which milk components, as compared to coconut water, were directly related to morphogenesis acceleration, since pupae were mainly generated from first instars in an average of only 7 days in milks compared with the 16–18 days seen in the controls (tap water). To do so, we fractionated the whole milk into its pH 4.7-precipitable casein fraction. A new bioassay indicated that this phosphoprotein, after adjusting the pH back to 6.0, was the main stimulating factor from milk.

Bacterial microbiota can play a beneficial role, either as a normal mutualistic population in *Aedes* gut or freely developing in the culture media (as in the present cases with milk as food source). The growing larvae clearly have the ability to ingest and digest any bacterial microflora from the water environment. Additionally, *Aedes* gut bacterial microflora efficiently assist in the food digestion of mosquitoes and in their full development and physiology (e.g., egg laying and fecundity) [34]. Gram-positive cocci (e.g., *Staphylococcus* spp.), as the second dominant bacterial population, were isolated from both larvae and pupae of *Ae. aegypti* [35]. *Streptococcus* spp., another Gram+ bacterial species, contaminates and ferments milk. [36].

A Thai study [37] used temporal temperature gradient gel electrophoresis for 16S rRNA population analysis in fourth instar *Ae. aegypti* larvae in a domestic water source, and demonstrated the presence of 24 bacteria taxa, including Proteobacteria, Firmicutes, Actinobacteria, and Bacteroides. Bacilli (acquired in the early stages of mosquito development) and Gammaproteobacteria were bacterial classes found in the mosquito larvae and infested/infected water samples, respectively. Bacilli are known chitinase producers [38].

As the milk samples utilized were probably aseptic, the bacterial population vigorously grown on them could be from the mosquito larvae themselves. Remember that for faster prospection of mosquito taxonomy (e.g., *Ae. aegypti* vs. *Ae. albopictus*; *Anopheles* spp.), larvae growth to adults is, by far, more efficiently and quickly achieved in milk, as shown in Figure 3, than employing procedures as daily replenishing water [39] or complex diets encompassing between three and fifteen ingredients [40].

## 5. Conclusions

This study allowed the acceleration of the morphogenetic cycle of *Aedes aegypti* with food additions such as coconut water and milk and its casein obtained by isoelectric precipitation, as well as yeast extract. Pursuing the goal to obtain the desirable instar IV larvae for subsequent bioassays of (phyto)larvicides, coconut water proved to be the more appropriate nutritional source for this specific purpose. Hence, we found satisfactory and innovative conditions to produce larvae more swiftly for larvicidal bioassays. Milk shortened the morphogenetic cycle and led to faster emergence of flying adults. Application of these nutrients (e.g., milk) can also help produce adult mosquitoes for taxonomic purposes if new outbreaks emerge, since treatments like population vaccination require exact identification of the mosquito vector. Our results also suggested that a combination of casein and coconut water may further accelerate the *Aedes* cycle, and this specific focus is currently being studied.

## Figures and Tables

**Table 1:** Nature and concentration of the nutritional sources.

Food supply	Quality features	Bioassay concentration (*μ*g/mL)
Milk WM	Whole	1.4 mg/mL
Milk LM	Reduced fat	1.4 mg/mL
Milk LZM	Lactose-free	1.4 mg/mL
Soymilk (SM)	Macchiato taste	1.4 mg/mL
Casein	Milk isolate	1.4 mg/mL
Coconut water (CNW)	Fresh, sterile	6 *μ*g/mL
Glycerol (G)	Lab reagent	16.6 *μ*g/mL
Yeast extract (Y)	Lab reagent	16.6 *μ*g/mL
Glycerol + yeast extract (GY)	Lab reagents	16.6 + 16.6 *μ*g/mL
Aminated carbohydrates	Lab reagents	2 mg/mL

## Data Availability

The photographs from Figures 1, 2, and 4 and histogram graphics from Figures 3 and 5 as data used to support the findings of this study are included within the article and are freely available after the publication of this paper.
